# A Consensus Framework for the Humanitarian Surgical Response to Armed Conflict in 21st Century Warfare

**DOI:** 10.1001/jamasurg.2019.4547

**Published:** 2019-11-13

**Authors:** Sherry M. Wren, Hannah B. Wild, Jennifer Gurney, Mohana Amirtharajah, Zachary W. Brown, Eileen M. Bulger, Frederick M. Burkle, Eric A. Elster, Joseph D. Forrester, Kent Garber, Richard A. Gosselin, Reinou S. Groen, Gary Hsin, Manjul Joshipura, Adam L. Kushner, Ian Norton, Inga Osmers, Heather Pagano, Tarek Razek, Jesús-Manuel Sáenz-Terrazas, Lilli Schussler, Barclay T. Stewart, Abd Al-Rahman Traboulsi, Miguel Trelles, John Troke, Christopher A. VanFosson, Paul H. Wise

**Affiliations:** 1 Stanford University School of Medicine, Stanford, California; 2 US Army Institute of Surgical Research/Joint Trauma System, San Antonio, Texas; 3 Médecins Sans Frontières, Amsterdam, the Netherlands; 4 Department of Surgery, Uniformed Services University, Bethesda, Maryland; 5 Department of Surgery, University of Washington, Seattle; 6 Committee on Trauma, American College of Surgeons, Chicago, Illinois; 7 Harvard T. H. Chan School of Public Health, Harvard Humanitarian Initiative, Harvard University, Cambridge, Massachusetts; 8 Department of Surgery, University of California, Los Angeles; 9 Orthopedic Department, University of California, San Francisco; 10 Department of Obstetrics and Gynecology, Alaska Native Medical Center, Anchorage; 11 Academy of Traumatology, Ahmedabad, India; 12 Center for Humanitarian Health, Johns Hopkins Bloomberg School of Public health, Baltimore, Maryland; 13 Emergency Operations and Partnerships, Emergency Operations, World Health Organization, Geneva, Switzerland; 14 Centre for Global Surgery, McGill University, Montreal, Quebec, Canada; 15 International Committee of the Red Cross, Geneva, Switzerland; 16 Icahn School of Medicine at Mount Sinai, New York, New York; 17 Samaritan’s Purse, Boone, North Carolina

## Abstract

**Importance:**

Armed conflict in the 21st century poses new challenges to a humanitarian surgical response, including changing security requirements, access to patients, and communities in need, limited deployable surgical assets, resource constraints, and the requirement to address both traumatic injuries as well as emergency surgical needs of the population. At the same time, recent improvements in trauma care and systems have reduced injury-related mortality. This combination of new challenges and medical capabilities warrants reconsideration of long-standing humanitarian surgery protocols.

**Objective:**

To describe a consensus framework for surgical care designed to respond to this emerging need.

**Design, Setting, and Participants:**

An international group of 35 representatives from humanitarian agencies, US military, and academic trauma programs was invited to the Stanford Humanitarian Surgical Response in Conflict Working Group to engage in a structured process to review extant trauma protocols and make recommendations for revision.

**Main Outcomes and Measures:**

The working group’s method adapted core elements of a modified Delphi process combined with consensus development conference from August 3 to August 5, 2018.

**Results:**

Lessons from civilian and military trauma systems as well as recent battlefield experiences in humanitarian settings were integrated into a tiered continuum of response from point of injury through rehabilitation. The framework addresses the security and medical requirements as well as ethical and legal principles that guide humanitarian action. The consensus framework includes trained, lay first responders; far-forward resuscitation/stabilization centers; rapid damage control surgical access; and definitive care facilities. The system also includes nontrauma surgical care, injury prevention, quality improvement, data collection, and predeployment training requirements.

**Conclusions and Relevance:**

Evidence suggests that modern trauma systems save lives. However, the requirements of providing this standard of care in insecure conflict settings places new burdens on humanitarian systems that must provide both emergency and trauma surgical care. This consensus framework integrates advances in trauma care and surgical systems in response to a changing security environment. It is possible to reduce disparities and improve the standard of care in these settings.

## Key Points

**Question:**

What are consensus components of a framework for humanitarian surgical response in modern conflict zones?

**Findings:**

This survey study using responses from 35 participants in the Stanford Humanitarian Surgical Response in Conflict Working Group suggests that humanitarian responses include both care of traumatic injury and emergency surgical needs of the population. Lessons from civilian and military trauma systems as well as humanitarian settings were translated into a tiered continuum of response from patient presentation through rehabilitation.

**Meaning:**

Evidence suggests that modern trauma systems save lives but providing this standard of care in insecure conflict settings places new burdens on humanitarian systems; the framework presented herein integrates advances in surgical care to these environments.

## Teaser

This study provides consensus guidelines on humanitarian surgical response formulated by representatives from international government and nongovernmental organizations for military and civilian populations in combat sites.

## Introduction

Over the course of the Iraq and Afghanistan conflicts, coalition military service members have been less likely to die of their injuries than service members from any other time in recorded history. This unprecedented battlefield survival was the result of a purposeful commitment to coordinate advances in trauma care with immediate frontline intervention and rapid transport to facilities with escalating surgical capabilities. The survival rate of individuals after battlefield injury has more than doubled when compared with the Vietnam War.^1^

An essential component of this improved trauma capability has been the rapid stabilization of the injured and their managed evacuation through a tiered system of care, each with escalating and increasingly specialized medical capabilities. The US military’s commitment to such a system took the form of the Joint Trauma System, which coupled rapid tiered response with data registries, ongoing performance improvement, and the development and implementation of clinical practice guidelines.^2^ Advances were both technical (eg, tourniquets, tranexamic acid, and hemostatic dressings) and systems-based (eg, advanced care during transport).^2,3^ An integrated continuum of care from the point of injury (POI) through rehabilitation included the following levels:

Role 1–tactical combat casualty care, in which frontline personnel perform hemorrhage control, resuscitation, and airway protection measures proximate to the POI;
Role 2–time and distance gaps from the POI to definitive surgical care, which are bridged via forward surgical teams capable of providing damage control resuscitation and surgery before rapid evacuation;
Role 3–combat support hospitals, which provide the highest level of care in the conflict zone. After stabilization, casualties are moved out of the country to definitive care facilities; and
Role 4–well-resourced definitive care facilities, which are located away from the conflict zone.

The successes of military trauma systems demonstrate the potential of advanced trauma management, and many of these advances have been successfully adopted into civilian trauma settings.^4^ In contrast, the humanitarian response in conflict settings not only has to provide trauma care but also emergency surgical care to the affected population. Providing humanitarian care in insecure environments poses major pragmatic and ethical challenges. Protracted intrastate conflicts that use siege warfare, the use of explosive weapons in densely populated urban settings, and the targeting of health facilities and other essential infrastructure (eg, water systems, food supply chains) can cause high levels of civilian mortality.^5,6^ This context also poses obstacles to the establishment of advanced trauma systems by humanitarian actors. Of particular concern are the positioning of humanitarian medical personnel close to the front lines, the control of evacuation pathways for casualties, secure supply chains, adequate human resources, and most fundamentally, the safety of patients and humanitarian personnel.

Several events have underscored the need to revise humanitarian protocols. The 2010 earthquake in Haiti exposed the inadequate training and coordination of emergency medical personnel, which resulted in care that has been described as “medically shameful.”^7^ Partly in response to this event, in 2013, the World Health Organization published what is called the Blue Book, a set of guidelines that established a framework of overarching standards for teams operating in sudden-onset disasters.^8^ Similar guidelines for the humanitarian response to conflict do not currently exist. The health response to the Battle of Mosul, Iraq, in 2016-2017 raised serious concerns regarding the deployment of humanitarian medical personnel under the direct protection of Iraqi security forces.^9^ The Mosul experience and the subsequent fighting in Raqqa, Syria, also raised concerns regarding military-civilian coordination and the responsibilities of militaries for the evacuation and care of civilian casualties.

While responsibility for frontline care of the wounded should rest first with the parties to the conflict,^10^ humanitarian organizations increasingly have become the major providers of medical care in areas of violent conflict. Humanitarian response to conflict is dissimilar from disaster and current World Health Organization Blue Book guidelines do not give adequate guidance. Unarmed humanitarian medical groups must operate in insecure environments and personnel must also adhere to the principles of humanity, neutrality, impartiality, and independence—requirements that place special demands on humanitarian systems.^11,12^ Civilian population needs differ from those of military personnel. Moreover, the military focus on frontline stabilization and rapid evacuation of patients is particularly difficult to implement in humanitarian settings. Unless these challenges are addressed, these dynamics can lead to high rates of preventable death and disability.

In response to these concerns, the Stanford Humanitarian Surgical Response in Conflict Working Group was established on March 20, 2018, to reevaluate the humanitarian medical response to conflict. The Group’s deliberations culminated in an in-person convening from August 3 to August 5, 2018, which produced a consensus framework for humanitarian trauma systems in conflict settings. The framework’s goal was to translate the progress made by trauma systems to the pragmatic realities of humanitarian medical provision in areas of violent conflict.

## Methods

Thirty-five participants were invited to the Stanford Humanitarian Surgical Response in Conflict Working Group. Contributors were selected on the basis of sector expertise, organization leadership, publication record in trauma care, trauma systems development, and humanitarian surgical care. This multinational group included senior representatives from civilian and military trauma systems, international humanitarian nongovernmental organizations, such as Médecins Sans Frontières, Samaritan’s Purse, International Committee of the Red Cross, World Health Organization, and academic trauma system and research centers. The group’s charge was to design a consensus framework for a new model for surgical humanitarian response, translating advances in combat casualty care to the modern humanitarian context. The first iteration of this framework was designed for protracted urban conflict, such as that seen in Syria and Iraq, with the understanding that this schema could be adapted to different humanitarian contexts in the future.

The working group’s process incorporated core elements of a modified Delphi process combined with formal consensus development methods with iterative rounds of inclusive, anonymized input leading to a transparent derivation of final decisions.^13^ Group members received a compilation of current protocols, resource lists, and relevant health services research on humanitarian and military trauma care in conflict settings. Documents were circulated electronically for feedback and voting. At each stage, input was synthesized and incorporated into revised documents that were recirculated for review. Members had the opportunity to comment, dissent, and revise their opinion after viewing the blinded results of others’ feedback. In addition, the group organizer prepared a final set of documents based on all feedback that served as the basis for discussion at the in-person convening.

From August 3 to 5, 2018, a 3-day, in-person convening occurred at Stanford University, Stanford, California. The proceedings consisted of presentations on the perspectives and practices of the represented organizations, as well as small-group design activities and entire-group discussion for transparent consensus building. No round proceeded until the objections of all members had been solicited, addressed, and resolved through discussion and consensus voting, and formal consensus had been achieved.^14^ The Stanford University Institutional Review Board determined that this research does not involve human subjects and therefore waived the need for informed consent.

### Humanitarian Response: Technical Framework

The objective of this framework is to provide a standardized approach to minimize preventable death and disability of people with surgical needs during conflict. This population includes patients with conflict-related violent injury as well as other surgical needs, such as obstructed labor, soft-tissue infections, nonviolent trauma, and intra-abdominal emergencies. Similar to the Joint Trauma System, this proposed framework consists of an integrated tiered chain of care with continuity from POI to definitive treatment and rehabilitation as well as systemwide advances (eg, injury prevention, data collection, and quality improvement).

## Results

### Level 1: Community First Responders

Expansion of tactical combat casualty care to all service members enabled the US military to decrease preventable prehospital deaths.^15^ In the humanitarian context, training civilian first responders should minimize responder risk and maximize opportunities to improve POI care. Basic skills should include (1) safe scene management (eg, removing responders and casualties from dangerous situations), (2) airway protection through jaw-thrust and chin-lift maneuvers and lateral trauma position for transportation,^16^ (3) bleeding control with manual compression and wound packing, (4) protecting the injured from the environment, and (5) rapid transportation of the injured to care. Bystander tourniquet application is not recommended, as transport may be prolonged and misapplied tourniquets may be ineffectual or harmful.^17^ Spine immobilization should also be avoided given prolonged transport times, reliance on makeshift transport, potential loss of airway, and difficulty managing complex spinal injury in this context.^18^ Interventions for cardiopulmonary arrest are not recommended, as individuals with no pulse do not survive in this environment.

More advanced training of a subset of community first responders could improve trauma outcomes in low-resource settings.^19,20^ Trauma first responders would complete identical training of the basic community responders and receive advanced training in (1) triage including identification of individuals who died or had injuries incompatible with life, (2) basic fracture immobilization, (3) preparation of casualties for transport, and (4) logistical options for transport adapted to the local context. In addition, trauma first responders ideally should provide ongoing community education and conflict first-aid training.

### Level 2: Trauma Stabilization Point

The trauma stabilization point (TSP) is proposed as the first site of care staffed by trained medical (not surgical) personnel. The TSP’s primary function is to provide far-forward emergency resuscitation and stabilization and must be capable of functioning in resource-constrained environments. The objective at this site is to control hemorrhage, manage airway emergencies, and initiate timely transfer to a higher level of care.^21^ Surgical procedures are not performed at this site.

By moving medical capabilities as close to the POI as possible, the TSP represents a significant change from most humanitarian responses. The role and location of the TSP must be continuously reassessed in light of conflict dynamics and security considerations. The utility of TSPs may be limited in certain contexts, particularly where fighting is sporadic or front lines are poorly defined. The potential contribution of the TSP is contingent on the presence of a system of care meeting the following requirements: (1) adequate transport, (2) capability to maintain care en route, and (3) transfer to a receiving facility able to provide more-advanced care.

The TSP would be the first site of medical triage influenced by patient injury and use of resources. The reciprocity between hemorrhage control and triage has been well recognized in the military trauma system of care. Life-saving measures for survivable injuries prioritizing circulation and control of massive hemorrhage with nonsurgical management of airway emergencies have been reported to decrease preventable deaths.^22^ Early hemorrhage control allows patients to survive; in addition, the triage category can be downstaged, as in the example of extremity hemorrhage.

Based on military data,^23^ TSPs would ideally be located within 10 minutes of the POI. Given contextual constraints, the goal should be within 20 minutes. Therefore, rapid patient evacuation is an essential component of the TSP. The TSP services should include (1) hemorrhage control via tourniquets and/or placement of a pelvic binder; (2) resuscitation, including tranexamic acid administration, intravenous and intraosseous line placement, and crystalloid infusion; (3) initial management of chest injury causing airway or circulatory compromise via decompression of the pleural space, application of chest seals, and insertion of pleural drains; (4) initial management of life-threatening infection via resuscitation and antibiotic administration as well as wound irrigation and debris removal; and (5) pain control. Owing to poor long-term prognosis, resuscitation of pulseless patients and emergency resuscitative thoracotomy should not be performed. The advisability of initiating invasive procedures at this site is contingent on the ability to sustain care beyond the TSP. Only patients requiring a higher level of care should be transferred to the definitive facility. The TSP is not intended to serve as a site of primary care or management of nontraumatic medical or surgical needs. In addition, definitive management and discharge of patients with non–life-threatening injury should be provided at the TSP, including acute wound and closed fracture management, pain management, and tetanus prevention. By consensus, context-specific recommendations for TSP personnel, which is a controversial subject,^13^ fell outside the group’s mandate.

Early component or whole blood transfusion has been reported to improve survival in patients with hemorrhagic shock and is the equalizer for long transport times to a higher level of care.^24^ Transfusion capability, which may be possible through walking blood banks, would augment the TSP. However, although desirable, blood transfusion at the TSP is currently not available in most settings.

### Level 3 Framework

#### Definitive Care Facility

After stabilization at the TSP, patients in need of additional care should be transported to a definitive care facility. This site would be the first point at which surgical care will be provided and must be capable of treating injuries as well as emergency surgical and obstetrical conditions. These facilities require significant investment in material and human resources to provide quality care. Minimum essential procedures provided at this site are presented in Box 1.

Box 1. **Level 3 Definitive and Contingency Care Facility and Advanced Care Capabilities**

**Definitive Care Facility**

- Imaging: radiography and ultrasonography
- Fracture management (open and closed)
- Management of spinal cord or column injuries
- Definitive surgical burn care
- Operative treatment of abdominal and obstetrical surgical emergencies
- Open skull fracture management
- Amputation and fasciotomy
- Herniorrhaphy (nonelective, primary tissue)
- Emergency genitourinary procedures
- Complex wound closure and skin graft
- Enucleation
- Operative management of fractured mandible
- Local or rotational flap
- Definitive vascular reconstruction
- Complex surgical treatment of wounds and infection
- Regional, spinal, and general anesthesia with intubation
- Transfusion services
- Complex perioperative care
- Intermediate level of care above ward
- Postanesthesia care unit
- Palliative care (eg, high-mortality burns)

**Contingency Facility^a^**

- Imaging: ultrasonography
- Diagnostic peritoneal lavage or aspiration
- Closed fracture management
- Conservative management of spinal cord or column injuries
- Emergency burn escharotomy and acute burn management
- Basic neck exploration
- Suprapubic tube placement
- Temporary vascular stabilization including shunt
- Open skull fracture management
- Amputation and fasciotomy
- Operative treatment of abdominal and obstetrical surgical emergencies
- Surgical treatment of wounds and infection
- Regional, spinal, and general anesthesia with intubation
- Postanesthesia recovery in operating suite
- Basic transfusion services
- Pain and symptom management

**Advanced Capability Package^b^**

**Medical Care**

- Ventilators
- Intensive care unit nursing staff
- Central venous catheters
- Cardiac monitoring
- IV inotropes

**Surgical Services**

- Cardiothoracic
- Neurosurgical
- Reconstructive

^a^This facility would provide damage control surgery en route to definitive surgery as needed.

^b^An advanced capability package can be used to increase the complexity and scope of surgery performed in the definitive care facilities by providing specialist surgeons and critical care services.

#### Contingency Facility

An innovation proposed by this group is the contingency facility. This facility should provide damage control surgery en route to definitive care as needed. Situations dictating the need for a contingency site include (1) the pace and volume of incoming casualties, (2) anticipated surge in casualties, (3) ability to provide timely damage-control surgery, and (4) other threats to the survival gains made at the TSP. Patients with critical status (ie, triage category red)^25^ should optimally receive surgical care within 45 minutes but no longer than 60 minutes after leaving the TSP. For those with less-critical conditions (ie, triage category yellow), this timeline extends to surgery within 4 hours optimally but no more than 6 hours. Although these timelines are ambitious in insecure environments, similar transport times have been achieved in other resource-constrained settings.^26^ Emergency abdominal and obstetric procedures could also be done at this site if timely transfer to the definitive site is not possible within a time frame that maximizes survival (Box 1). Contingency facilities must be mobile and, as at the TSP, stringent triage criteria must be used given finite resources and the inability to provide mechanical ventilation while awaiting transport.

#### Advanced Capability Package

Another proposed innovation is a standardized advanced capability package that can be used by definitive care facilities to provide higher-level specialty surgical and critical care. The advanced capability package would consist of personnel with specialized surgical training (eg, cardiothoracic, neurologic, plastic surgeons) and equipment (eg, mechanical ventilation, central venous catheters, advanced monitoring, specialized surgical equipment), which could be integrated into the core platform as circumstances permit.

### Level 4: Reconstruction and Rehabilitation

Despite resource limitations, the system should seek to improve functional outcomes by including basic reconstructive and rehabilitative services (Table 1). However, the complexity of reconstructive care should not exceed contextual realities in terms of hygiene, required skill, nursing, physiotherapy, postoperative care capabilities, and outpatient or community rehabilitation services. The decision to perform advanced reconstructive procedures must weigh the risks of potential complications. Early reconstruction should prioritize procedures that optimize functional outcomes, emphasizing physiotherapy and rehabilitation as integral components of the process.^27^

**Table 1.** Reconstructive and Rehabilitative Services

Level	Procedure
Minimum	Burn reconstruction, including skin grafts and contracture release Local flaps for soft-tissue coverage Stump revision and provision of postamputation care Reanastomosis of bowel stoma Treatment of acute osteomyelitis Physiotherapy, including provision of crutches, walking frames Conservative management of spinal cord/column injuries Mental health services
Context dependent	Internal fixation of fractures
Not to be performed (nonexhaustive)	Cosmetic surgery Dental reconstruction Complex congenital disorders Free flaps Arthroplasty Repair of obstetrical fistula Limb lengthening/bone transport

A significant percentage of patients who experience blast and explosion injuries require amputation. It is critical for surgeons to consider function in the creation of the residual limb. Of equal importance is training in adaptive devices, safe transfer techniques, and physiotherapy for residual limb preparation. When feasible, the provision of adequate prostheses would permit patients to regain additional function. At present there are few organizations with the means to provide and maintain prosthetics in these settings. Efforts should be made to establish international networks and allocate funds for prosthesis provision and follow-up.

Burn injuries are common in both conflict and low-income contexts. Therefore, acute burn care, reconstruction, and rehabilitation are critical functions to prevent death and disability. Mental health services should be an integral part of care and follow-up planning at acute care facilities. In addition, reconstruction of congenital disorders and other complex non–conflict-related conditions should be deferred during active conflict given resource constraints.

### Systemwide Requirements

In addition to the technical requirements and competencies necessary at each level of care, there are features that should be integrated within the system to optimize care. These features include predeployment education, injury prevention, data collection, and quality improvement.

#### Humanitarian Principles and Context

The ability for humanitarian organizations to provide surgical care proximate to the time of injury is complicated by security and access constraints.^28,29^ Humanitarian medical teams are unarmed and may come under fire if operating near the front line or, increasingly, are directly targeted. Attempts to reconcile the dual imperatives of safety and rapid medical intervention have proven controversial.^30^ Placing humanitarian medical personnel under the protection of combatant forces challenges the long-standing humanitarian principles of neutrality and independence, which are in part designed to enhance humanitarian access and safety.^11^ Although these tensions are unresolved, it remains important to distinguish humanitarian activities from other forms of assistance associated with “interest-conflicted” parties, which may include the host state. Efforts to document attacks on medical personnel and/or zones established to care for the wounded and sick and hold perpetrators accountable should be strongly supported.^31,32^

Given the importance and complexity of these issues, all medical personnel serving in conflict areas should have basic training in humanitarian ethics and/or medical ethics specific to the setting or armed conflict, international humanitarian law, and knowledge of the local sociocultural and political context.^33^ Special attention should be paid to the principle of impartiality, which requires that assistance be provided based on medical need regardless of a patient’s political affiliation or preinjury combatant status.^11^ Counter-terrorism strategies can conflict with medical impartiality, and potential conflicts should be managed with an understanding of international humanitarian law and informed anticipation of these pressures. Standardized, pragmatic educational materials for medical personnel and predeployment checklists may prove useful (Box 2).

Box 2. **Predeployment Minimal Training Standards: Humanitarian Principles and Context**

- Training in medical ethics, humanitarian principles, and international humanitarian law
- Training in international and domestic laws governing medical action and patient protections
- Context briefing on the local setting, including cultural and political dynamics
- Security protocols and orientation to operations in high-risk environments, kidnapping, handling of unexploded ordnance and nuclear, biological, and chemical defense procedures
- Code of professional conduct/behavioral commitments
- Policies for media relations, including the ethical use of social media

#### Injury Prevention

Injury prevention may provide additional opportunities to reduce preventable death and disability (Table 2).^34^ Prevention measures should include efforts to anticipate and mitigate risks associated with conflict, including timely escape, avoidance of landmines and unexploded ordnance, and safety around damaged infrastructures.^35^ First responders, whether lay or professional, should have training in safe recovery of the wounded, use of personal protection, risk reduction strategies, and contingency planning. Prevention programs should engage patients, individuals, community leaders, civil societies, nongovernmental organizations, and when appropriate, military stakeholders and/or governments. Use of local systems to disseminate information (eg, public service announcements via radio or social media, training of community health workers) may improve their reach. Population safeguards, such as early warning systems and egress assistance, may also prove to be useful.

**Table 2.** Functions and Representative Activities for Injury Prevention in Conflict Settings

Function^34^	Representative Activities
Strengthen individual knowledge and skills	Landmine/unexploded ordnance risk reduction campaign Burn and electrical injury prevention Instruction on safe management of structural hazards
Develop community awareness and engagement	Public service announcements Engagement with community health workers Train-the-trainer models for safety promotion
Educate providers	Injury risk screening and reduction in health facilities Awareness of gender-based violence signs and resources Safe recovery education for first responders
Foster coalitions	Interagency collaboration for prevention initiatives Coordination with existing injury prevention actors and campaigns
Population safeguards	Early warning systems and egress assistance
Advocate for safety	Mobilizing social support for specific prevention efforts Bringing forward/supporting relevant treaties

#### Data Collection and Quality Improvement

Advances in trauma systems development have been predicated on the ability to evaluate data from trauma registries such as the Department of Defense Trauma Registry and American College of Surgeons National Trauma Data Bank.^36,37^ A clinical data collection system is foundational for understanding the evolving epidemiology of surgical disease in conflict and for improving the quality of response.

The recommended minimum data that should be collected at each point of care and coordinated among all actors are outlined in Box 3.^38,39^ Desired data elements include the geographic place of injury, clinical complications, functional status at disposition and, if possible, measures of long-term patient outcomes. If multiple organizations are involved, responsibilities for data collection and analysis should be clearly defined and occur at consistent points throughout the system. The registration and transmission of data must use security safeguards to protect the identity of the patient, maintain patient safety, and link individual records across the continuum of care. To minimize the task burden and maximize utility, data collection should be completed electronically and include a limited number of elements at each site. In addition, systematic data analysis should be undertaken in real time to ensure immediate feedback and response refinement in a rapidly changing environment.

Box 3. **Minimum Data Set for Humanitarian Trauma System**

**Required**

Patient demographic information (sex, age)
Mechanism and anatomic location of injury
Estimated time of injury
Time of arrival at each point of care
Mode of transport to point of care
Vital signs
Triage category^25^ and Kampala Trauma Score^38^
Procedures performed
Disposition outcome (dead/alive)
Time of disposition
Intended destination at disposition

**Desirable**

Geographic place of injury (if not risking patient safety)
Clinical complications
Information about the patient’s basic functional status at disposition
Any available data about long-term patient outcomes

## Discussion

This framework presents minimum standards for humanitarian surgical response in areas beset by armed conflict. Even in high-risk security and resource-constrained conflict settings there exists a realistic potential to prevent death and disability. Adapting advances in trauma systems to the humanitarian context could also improve outcomes in the care of other surgical emergencies.

The implementation of trauma systems has consistently reported reduction of preventable death and disability in both military as well as civilian environments.^40,41^ This framework builds on this evidence, optimizing rapid intervention to manage the leading causes of death, hemorrhage, and airway compromise. Military and civilian communities have approached these challenges by developing coordinated systems of trauma care that match the nature and urgency of patient needs with provision of escalating surgical capabilities. Communication between system components is also essential to facilitate clinical standards, transitions of care, data collection, and quality improvement.

### Limitations

There are significant challenges to the implementation of this framework; however, its utility is based on providing a basic blueprint that can be adapted for different conflict settings. The Stanford Working Group also identified areas for further consideration, including (1) standardized technical and contextual training for personnel deployed to conflict settings, (2) development of a uniform data registry with embedded quality improvement capabilities, and (3) a system for communication and coordination of care between all actors working in the zone of conflict, as collaboration between all medical actors is critical to the success of a surgical care system.

## Conclusions

Ideally, the principles used to establish military and civilian trauma systems should be applied to the humanitarian care of patients in conflict zones. Organized trauma systems save lives and have become the standard of care; as such, it is vital for all actors to extend these benefits to populations in conflict.
